# Network-Based Study Reveals Potential Infection Pathways of Hepatitis-C Leading to Various Diseases

**DOI:** 10.1371/journal.pone.0094029

**Published:** 2014-04-17

**Authors:** Anirban Mukhopadhyay, Ujjwal Maulik

**Affiliations:** 1 Department of Computer Science and Engineering, University of Kalyani, Kalyani, West Bengal, India; 2 Department of Computer Science and Engineering, Jadavpur University, Kolkata, West Bengal, India; Temple University School of Medicine, United States of America

## Abstract

Protein-protein interaction network-based study of viral pathogenesis has been gaining popularity among computational biologists in recent days. In the present study we attempt to investigate the possible pathways of hepatitis-C virus (HCV) infection by integrating the HCV-human interaction network, human protein interactome and human genetic disease association network. We have proposed quasi-biclique and quasi-clique mining algorithms to integrate these three networks to identify infection gateway host proteins and possible pathways of HCV pathogenesis leading to various diseases. Integrated study of three networks, namely HCV-human interaction network, human protein interaction network, and human proteins-disease association network reveals potential pathways of infection by the HCV that lead to various diseases including cancers. The gateway proteins have been found to be biologically coherent and have high degrees in human interactome compared to the other virus-targeted proteins. The analyses done in this study provide possible targets for more effective anti-hepatitis-C therapeutic involvement.

## Introduction

Hepatitis-C virus (HCV) causes the infectious disease Hepatitis-C which primarily affects the liver. It is important to identify the potential target human proteins that lead to different diseases caused by hepatitis-C virus infection. Analyzing the regulation between viral and host proteins in different organisms helps to uncover the underlying mechanism of various viral diseases. Protein-protein interaction (PPI) information provides a local as well as a global view of the interaction modules of proteins participating in similar biological activities. Such interaction information can be obtained via biological experiments or can be predicted using computational approaches [1]. Among the experimental methods, yeast two-hybrid (Y2H) screens have been widely used by the biologists. The Y2H system can detect both transient and stable interactions. The works in [2] and [3] deal with the identification of PPIs in *Saccharomyces cerevisiae* using yeast two-hybrid screens. The Y2H approach has also been utilized in the analysis of human PPIs in some earlier studies [4], [5]. Another popularly used experimental method in the context of PPI is mass spectrometry which is used to identify the components of protein complexes. Use of mass spectrometry method for detecting PPIs can be found in [6], [7].

One of the main goals in research of PPI is to predict possible viral-host interactions. This interaction information can be utilized to identify and prioritize the important viral-host interactions. This is specifically aimed at assisting drug developers targeting protein interactions for the development of specially designed small molecules to inhibit potential HCV-Human PPIs. Targeting protein-protein interactions has relatively recently been established to be a promising alternative to the conventional approach to drug design [8], [9].

Although there have been many studies on determining and analyzing PPIs in a single organism, not much work can be found on computational analysis of viral-host interactions. In very recent times, some computational analysis of viral-host interactions, specially in HIV-1-human PPIs [10]–[15] have been done. Some recent studies have analyzed the viral-host interactions for some individual HCV proteins. For example, in [16], a study on NS2 protein of HCV is conducted and its role in HCV life cycle is discussed. In [17], the interactions of HCV proteins CORE and NS4B with human proteins have been analyzed for understanding the biological context in HCV pathogenesis. In [18], the authors have revealed that the HCV protein NS2 interacts with different structural and non-structural proteins for virus assembly. In another work [19], an integrative network analysis is performed to identify key genes and pathways in the progression of hepatitis C virus induced hepatocellular carcinoma. However, no global system-wide study based on the HCV-human interaction network is available in literature. Motivated by this, in the present work, the PPI records between HCV proteins and human (*Homo sapiens*) proteins reported in a recently published dataset [20] are collected. This interaction information, all together, can be visualized as a bipartite graph, where two sets of nodes denote HCV proteins and human proteins, respectively, and the edges denote the interactions. In this work, the bipartite network is mined to identify the strong interacting modules, which are effectively quasi-bicliques. We further extend the study by clustering the human protein-protein interaction network to identify the possible quasi-cliques that overlap with the quasi-bicliques identified in the previous step. The human proteins participating in these quasi-cliques are considered as gateways of infection and are further investigated for their functional characteristics. Subsequently, the bipartite network representing the association of human proteins with various disease types is mined to find possible quasi-bicliques that overlap with the gateway proteins discovered in the previous stage. Thus we explore three networks, namely, HCV-human interaction network, human protein interaction network, and human proteins-disease association network globally to discover the potential pathways of infection by the HCV viruses that lead to various diseases including cancers. The analyses done in this study may provide possible targets for more effective anti-hepatitis-C therapeutic involvement.

## Materials and Methods

In the present study, three different networks are mined. First one is the HCV-human protein interaction network. This network is modeled as a bipartite graph with two sets of nodes, one set corresponding to the HCV proteins and the other set corresponding to the human proteins. The edges represent presence of interactions between the corresponding HCV and human proteins. The second network is human protein interaction network, which is modeled as a graph. Nodes represent the human proteins and the edges represent interactions among them. The third network represents the associations between human proteins and disease. Hence this disease association network is also modeled as a bipartite graph with two sets of nodes representing human proteins and diseases, respectively. The edges of this graph represent the association of the human proteins with diseases.

Before describing the proposed methods, here we first define a few terms to help subsequent discussions 21,22.

**Definition 1** (Graph). The term graph is used throughout to denote an unweighted and undirected simple graph (without self-loops or parallel edges) 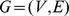, where *V* and *E* are the vertex and edge sets, respectively. Here *E* is represented as a set of vertex-pairs, i.e., 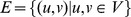.

**Definition 2** (Degree of a Vertex). The degree of a vertex *v_i_*, denoted as *d*(*v_i_*) in a graph, is said to be the number of edges incident to it. Hence 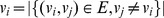.

A graph 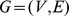 may contain subgraphs. A clique is a complete subgraph of a graph.

**Definition 3** (Clique). A subgraph 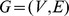 is said to be a clique if for each vertex pair 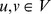, there is an edge 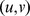.

As can be seen, the edge set *E* of a clique can readily be obtained from the vertex set *V*, and therefore a clique may be simply denoted as *G* = *V*.

**Definition 4** (*γ*-quasi-clique). In a graph 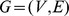, a subgraph 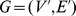, 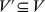, 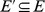, is said to be a *γ*-quasi-clique 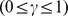 if the subgraph induced by this set of vertices contains at least 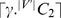 edges.

We denote the cardinality of a vertex set *V* as |*V*|. A graph is bipartite if its vertex set can be distinguished into a pair of partitions. It is formally defined as follows.

**Definition 5** (Bipartite graph). A graph 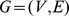 is said to be bipartite if its vertex set *V* can be partitioned into two nonempty and disjoint sets *V*_1_ and *V*_2_ such that 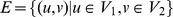.

Therefore, a bipartite graph 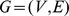 can also be represented as 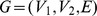. As the graphs may have subgraphs, bipartite graphs may also contain subgraphs. A biclique is a complete bipartite subgraph.

**Definition 6** (Biclique). A bipartite subgraph 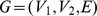 is said to be a biclique if for each vertex pair 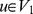 and 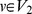, there is an edge 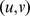.

As can be seen, the edge set *E* of a biclique can be readily obtained from the two vertex sets 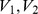, and therefore a biclique may be simply denoted as 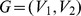.

**Definition 7** (*γ*-quasi-biclique). In a bipartite graph 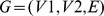, a bipartite subgraph 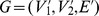, 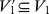, 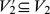, 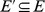, is said to be a *γ*-quasi-biclique 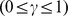 if the subgraph induced by these two sets of vertices contains at least 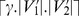 edges.

**Figure 1:**
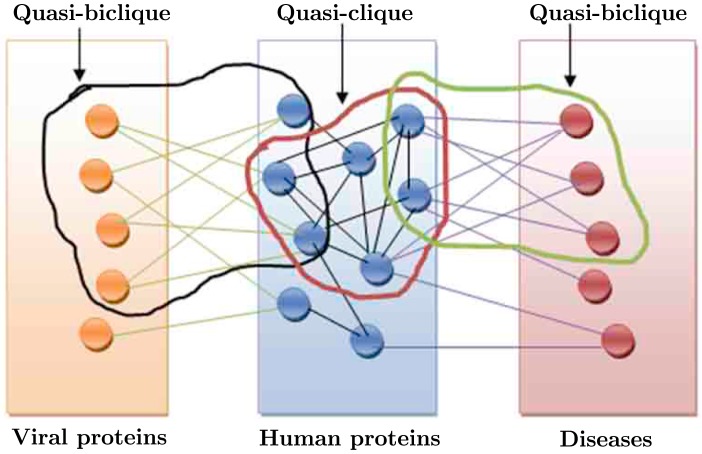
The diagrammatic representation of the proposed study. The orange circles represent the HCV proteins. The blue circles represent the human proteins. The pink circles represent the diseases. The green edges represent the interaction between HCV proteins and human proteins. The black edges represent the interactions among human proteins. The violet edges represent the associations between human proteins and diseases. The quasi-bicliques and bicliques are shown also. The quasi-biclique in the HCV-human bipartite network overlaps with the quasi-clique in the human protein interaction network. The quasi-clique in the human protein interaction network overlaps with the quasi-biclique in the human protein-disease association network.

The proposed study consists of three stages. First we mine strong *γ*-quasi-bicliques from the first bipartite graph that represents the interactions between viral and human proteins. The obtained quasi-bicliques are strong interaction modules consisting of the HCV and human proteins. Thereafter, in the second stage we cluster the human protein-protein interaction network to identify the possible strong *γ*-quasi-cliques that overlap with the quasi-bicliques identified in the previous step. The human proteins participating in these quasi-cliques are considered as gateways of infection and are further investigated for their functional characteristics. Subsequently, the bipartite network representing the association of human proteins with various disease types is mined to find possible strong *γ*-quasi-bicliques that overlap with the gateway proteins discovered in the previous stage. Hence we explore three networks, namely, HCV-human interaction network, human protein interaction network, and human proteins-disease association network globally to discover the potential pathways of infection by the HCV viruses that lead to various diseases including cancers. Fig. 1 diagrammatically demonstrates the study conducted in this article.

In this article we have proposed an algorithm based on hierarchical clustering that can mine both *γ*-quasi-cliques and *γ*-quasi-bicliques from graphs and bipartite graphs, respectively. The algorithm is basically a quasi-clique mining algorithm, however, with a little modification, this can also be used to mine quasi-bicliques as well. First we describe the algorithm for mining quasi-cliques from a graph. Thereafter, how this algorithm is modified to mine quasi-bicliques is described below.

### Mining *γ*-Quasi-Cliques

The proposed algorithm for mining *γ*-quasi-cliques is based on hierarchical average linkage clustering method [23], [24]. Given an input graph 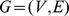, first the shortest path distances (number of edges) between all pairs of vertices are computed. Thereafter the dendrogram is built using agglomerative average linkage method. In this method, first a cluster is formed corresponding to each vertex of the graph. Thereafter two nearest vertices as per shortest path distance are combined to form a new cluster. This continues until there remains only one cluster containing all the vertices. The distance between any two cluster is computed as the average distance between all the vertices in the two clusters. The tree representing the hierarchical relationships among the clusters formed in this way is called the dendrogram.

After building the dendrogram, we start scanning from the top of the dendrogram to the bottom, one step at a time. Every time a cluster is divided into two, we examine the two clusters whether they are *γ*-quasi-cliques given a *γ* value. If any cluster satisfies this criterion, we do not further divide that cluster, i.e., the subtree rooted by this cluster is no more explored and this cluster is returned as one *γ*-quasi-clique. The clusters that are not *γ*-quasi-cliques are recursively divided as per the dendrogram until they provide some *γ*-quasi-clique, or reaches the threshold of quasi-clique size (minimum number of vertices to be present in the quasi-clique). Hence, the algorithm returns a set of maximal *γ*-quasi-cliques, i.e., the *γ*-quasi-cliques which are not completely included in another *γ*-quasi-clique.

### Mining *γ*-Quasi-Bicliques

The algorithm for mining *γ*-quasi-bicliques, which are equivalent to biclusters [25], is exactly same as mining *γ*-quasi-cliques, the only modification is done in the distance matrix. In this case also, we compute the shortest path between the nodes in the input bipartite graph 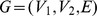. Note that here the distance between two vertices 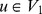 and 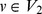 can be any odd value ≥1, since *u* and *v* may not be directly connected, but there may be a path between this two that contains a number of vertices from *V*_1_ and *V*_2_ in alternative positions. Any two vertices 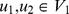 are never connected directly in a bipartite graph, however they may be connected through a set of vertices from *V*_2_ and *V*_1_ in an alternative fashion, and thus the distance between any two vertices in *V*_1_ is always an even value ≥2. Similar is the case for any two vertices in set *V*_2_.

In our study, The number of HCV proteins (set *V*_1_) is far more less than the number of human proteins (set *V*_2_). Therefore to increase the participation of HCV proteins in the *γ*-quasi-bicliques, we have modified the distance function between two viral proteins. In the modified version, the distance between any two viral proteins that are connected by a series of alternative human and viral proteins, i.e., which belong to the same connected component in the bipartite graph, is made 1. Thus the viral proteins that belong to the same connected component come closer to each other virtually and the number of viral proteins in the *γ*-quasi-cliques increases. The similar approach is adopted while finding the quasi-bicliques between the human proteins and diseases to increase the participation of the human proteins.

### Databases and Preprocessing

As stated before, we deal with three networks, namely, HCV-human PPI network, human PPI network and human protein-disease association network. In this section, the collection and preprocessing of the datasets have been described below.

### HCV-Human Protein Interaction Database

**Figure 2:**
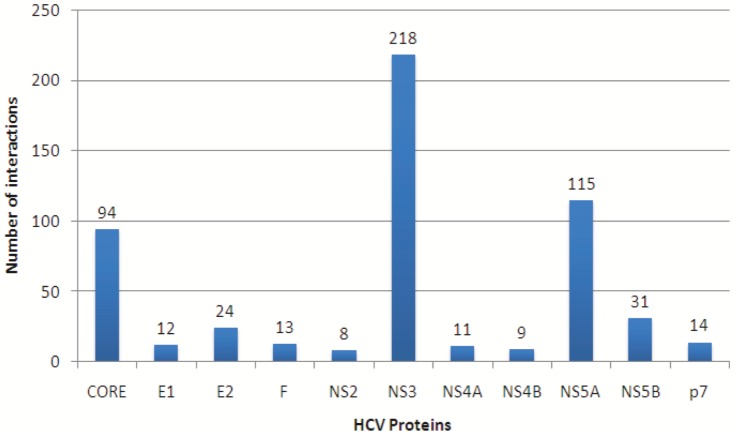
Distribution of interactions in the Hepatitis-C-Human bipartite interaction network with respect to the 11 HCV proteins. The HCV protein NS3 interacts with maximum number of human proteins (218), whereas NS2 is found to interact with minimum number of human proteins (8). Among the other HCV proteins, NS5A and CORE have reasonable number of interactions with the human proteins (115 and 94, respectively).

The protein interaction information between the HCV proteins and human proteins have been collected from a recently developed HCV-human protein interaction database called HCVpro [20] publicly available at http://cbrc.kaust.edu.sa/hcvpro/. This viral-host PPI database has been manually curated and it stores only those HCV-human PPIs that pass through a very strict filtering process [20]. Hence this repository maintains a very high-quality PPI information. It can be noted that there is another well-known and widely used database of hepatitis C-human protein interactions which is available at [26]. However, we found that the HCVpro database covers ∼94% of the interactions present in that database. Therefore we decided to use the newer database HCVpro. The HCVpro database contains the interactions among 11 HCV proteins (CORE, E1, E2, F, NS2, NS3, NS4A, NS4B, NS5A, NS5B, p7) and 455 human proteins. The total number of interactions is 549. The interactions are given in File S1. Fig. 2 shows the distribution of the interactions with respect to each of the HCV proteins. It is evident from the figure that the HCV protein NS3 interacts with maximum number of human proteins (218), whereas NS2 is found to interact with minimum number of human proteins (8). Among the other HCV proteins, NS5A and CORE have reasonable number of interactions with the human proteins (115 and 94, respectively). After removing the redundant interactions, the number of unique interactions reduces to 524. These 524 interactions among 11 HCV proteins and 455 human proteins are used for preparing the bipartite network between viral and host proteins and the maximal *γ*-quasi-bicliques are mined from this bipartite network as described in the previous section.

### Human Protein Interaction Database

The primary objective of mining human protein interaction database is to find *γ*-quasi-cliques that overlap with the *γ*-quasi-bicliques identified in the previous stage of the study. Hence to avoid huge computational complexity in mining quasi-cliques from the complete human protein interaction database, we concentrate only on the part of the human PPI that contains the human proteins present in the identified *γ*-quasi-bicliques in the previous stage. For this, the function protein association network STRING (http://string-db.org/) has been utilized. For each quasi-biclique identified in the previous stage, the participating human proteins are given as input to STRING and STRING generates an interactome containing these human proteins and other additional human proteins. We consider the predictions based on co-expression, experiments and databases only. We consider only the interactions with confidence of at least 0.8 (in a confidence scale between 0 and 1). This ensures that we consider only those PPIs that have reasonable number of evidences in literature. Maximum number of interactions per protein is set to 100. From the resultant PPI, the *γ*-quasi-clique mining algorithm described in previous section is applied to obtain any quasi-clique that overlaps the previously mined quasi-biclique on which the present human PPI has been built.

### Human Protein-Disease Association Database

**Figure 3:**
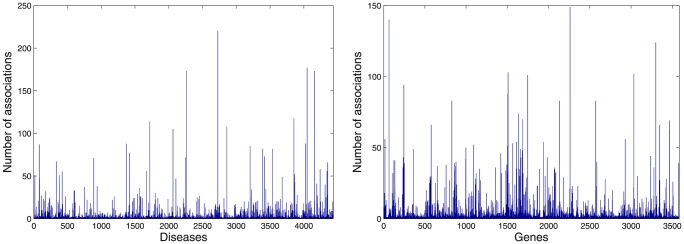
Distribution of associations in the human gene-disease association network. The left hand side figure shows the distribution of associations with respect to all the disease. The right hand side figure shows the distribution of associations with respect to all the genes.

The Genetic Disease Association Database [27] (http://geneticassociationdb.nih.gov/) archives the human genetic association studies on various types of complex diseases and disorders. The database contains summary data extracted from published articles in peer reviewed journals on candidate gene and GWAS studies. The database contains both positive (if the gene/protein is known to have association with the phenotype) and negative (if a gene/protein is known to have lack of association with the phenotype) associations, and also unknown (no specific information) associations. The network has been given in File S3. All the gene-disease association information have been downloaded from the database and the associations other than positive ones are filtered out. We found approximately 4200 unique diseases which are associated with approximately 3600 human genes/proteins, resulting approximately a total of 12400 unique gene-disease associations. In Fig. 3, we have demonstrated the distributions of associations with respect to both diseases and genes. In both cases, it can be noticed that only few diseases have association with many human proteins, but most of the diseases are associated with only a few human proteins. The density of this bipartite network in ∼0.0007 only, which indicates the sparseness of the network. The human proteins belonging to the quasi-cliques identified in the previous stage are considered and the bipartite network with these human proteins and diseases connected to them is formed. Thereafter, the *γ*-quasi-biclique mining algorithm is applied to this bipartite network to obtain the strong maximal quasi-bicliques from this network.

## Results and Discussion

In this section, we discuss the results of the proposed study.

### Mining Quasi-Bicliques in HCV-Human Protein Interaction Network

First we apply the proposed *γ*-quasi-biclique mining algorithm on the HCV-human protein interaction network collected from HCVpro. The value of *γ* has been set to 0.5. This is done as follows. We varied *γ* value from 0.1 to 0.9 with step size 0.1 and varied the minimum number of HCV proteins present in a quasi-biclique *n* from 2 to 5 with step size 1. For each combination of *γ* and *n* the algorithm is executed. In each case, the statistical significance of the set of resultant quasi-bicliques (if found) is investigated. To test the statistical significance of a quasi-biclique of size *x*×*y*, the bipartite graph is perturbed randomly 10,000 times (without changing the degrees of HCV proteins) and a quasi-biclique of size *x*×*y* is picked up randomly from the perturbed graph. Then we conduct the Wilcoxon ranksum test to find whether the density of the actual quasi-biclique is significantly better than the mean density of the random quasi-bicliques of same size. This returns a p-value and lower the p-value more significant is the quasi-biclique under consideration. For a combination of *γ* and *n* value, the average p-value over all the quasi-bicliques obtained is computed and we found that for *γ* = 0.5 and *n* = 3 the average p-value is minimum. Hence we set the *γ* value to 0.5 and quasi-bicliques having at least three HCV proteins (*n* = 3) are considered only. This results in two quasi-bicliques 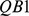 and 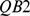, respectively. Different statistics about the two quasi-bicliques found are reported in Table 1. The densities (i.e., ratio of the maximum number of interactions present in the quasi-biclique to the maximum possible number of interactions) of the two quasi-bicliques obtained are 0.6786 and 0.5400, respectively. The first quasi-biclique consists of the HCV proteins CORE, NS3 and NS5A and 28 human proteins. Note that these three HCV proteins are the top three highest degree HCV proteins in the network. The other quasi-biclique consists of five HCV proteins E1, E2, NS2, NS4A and NS5B and 10 human proteins.

**Table 1:** Quasi-bicliques found from HCV-human protein interaction database.

Quasi-biclique	HCV proteins	Human proteins	Density
*QB*1	Count: 3	Count: 28	
	CORE, NS3, NS5A	EFEMP1, EIF2AK2, FBLN2, FBLN5, FTH1, HIVEP2, HNRNPK, JAK1, KPNA1, LTBP4, MAGED1, NAP1L1, NAP1L2, PSMB9, PSME3, RNF31, SMAD3, STAT1, STAT3, TBP, TLR2, TP53, TP53BP2, TRADD, TRAF2, TXNDC11, VIM, VWF	0.6786
*QB*2	Count: 5	Count: 10	
	E1, E2, NS2, NS4A, NS5B	CALR, CANX, CD209, CLEC4M, HOXD8, HSPA5, LTF, NR4A1, SETD2, UBQLN1	0.5400

The HCV proteins and human proteins involved in the quasi-bicliques are reported along with the densities of the quasi-bicliques.

### Mining Quasi-Cliques in Human Protein Interaction Network

**Figure 4:**
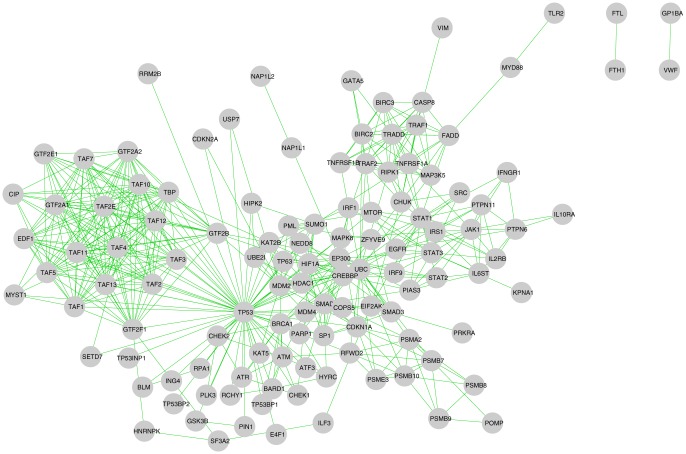
Human protein interactome induced by first quasi-biclique QB1. The interactome consists of 120 human proteins and 509 interactions among them. The density of the interactome is nearly 0.07.

In the next stage, as discussed before, the human proteins participating in the quasi-bicliques are given as the input to the STRING database. The human proteins involved in the first quasi-biclique 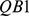 (Table 1) are first given to the STRING database with the parameter setting described in Section. This induces a human interactome consisting of 120 human proteins (Fig. 4 shows the interactome). Although this network is very sparse (density ∼0.07), a few denser regions are clearly visible from the figure. After applying the quasi-clique mining algorithm described before. The *γ* value is fixed to 0.6 and the minimum number of nodes allowed is set to 4. We obtained 9 dense quasi-cliques from the interactome. Out of these 9 quasi-cliques, 5 have overlaps with the first quasi-biclique discovered in the previous stage. Different statistics of these 5 quasi-cliques are shown in Table 2.

**Table 2:** Quasi-cliques found from human protein interactome that overlap with the human proteins involved in the first quasi-biclique of Table 1.

Quasi-clique	Human proteins	Density	Overlapping proteins with first quasi-clique
*QC*1	Count: 8		
	POMP, PSMA2, PSMB10, PSMB7, PSMB8, PSMB9, PSME3, RFWD2	0.6786	PSMB9, PSME3
*QC*2	Count: 14		
	BIRC2, BIRC3, CASP8, FADD, GATA5, MAP3K5, RIPK1, TNFRSF1A, TNFRSF1B, TRADD, TRAF1, TRAF2, UBC, VIM	0.6484	TRADD, TRAF2, VIM
*QC*3	Count: 23		
	CIP, EDF1, GTF2A1, GTF2A2, GTF2B, GTF2E1, GTF2F1, HNRNPK, MYST1, SETD7, SF3A2, TAF1, TAF10, TAF11, TAF12, TAF13, TAF2, TAF2E, TAF3, TAF4, TAF5, TAF7, TBP	0.6324	HNRNPK, TBP
*QC*4	Count: 8		
	HDAC1, HIPK2, MDM2, MDM4, SUMO1, TP53, UBE2I, USp7	0.6429	TP53
*QC*5	Count: 8		
	EGFR, IL6ST, JAK1, PIAS3, SRC, STAT1, STAT2, STAT3	0.7143	JAK1, STAT1, STAT3

The human proteins involved in the quasi-cliques are reported along with the densities of the quasi-cliques and the overlapping human proteins with the first quasi-biclique.

**Figure 5:**
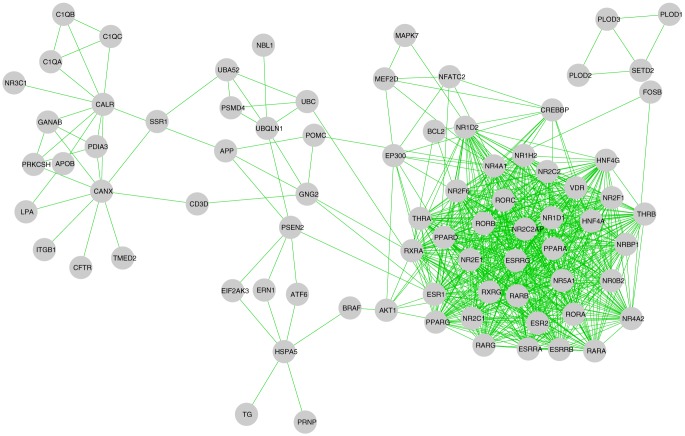
Human protein interactome induced by second quasi-biclique QB2. The interactome consists of 79 human proteins and 693 interactions among them. The density of the interactome is nearly 0.22.

After application of the quasi-clique finding algorithm on the interactome induced by the second quasi-biclique 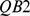 of Table 1, it provides 4 quasi-cliques that overlap this quasi-biclique. The interactome induced by the second quasi-biclique consists of 79 human proteins (This interactome has been shown in Fig. 5). This network has density of ∼0.22. However, here also, a few denser regions can be noticed from the figure. The 4 quasi-cliques as found by the algorithm have been reported in Table 3. It is evident from the table that these quasi-cliques overlap with the second quasi-biclique on only one human protein each. Both the human interactomes induced by quasi-bicliques 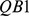 and 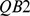 are reported in File S2. All the quasi-bicliques and quasi-cliques are reported in File S4.

**Table 3:** Quasi-cliques found from human protein interactome that overlap with the human proteins involved in the second quasi-biclique of Table 1.

Quasi-clique	Human proteins	Density	Overlapping proteins with second quasi-biclique
*QC*6	Count: 4		
	PLOD1, PLOD2, PLOD3, SETD2	0.8333	SETD2
*QC*7	Count: 5		
	NBL1, PSMD4, UBA52, UBC, UBQLN1	0.7000	UBQLN1
*QC*8	Count: 45		
	BCL2, CD3D, CREBBP, EP300, ESR1, ESR2, ESRRA, ESRRB, ESRRG, FOSB, GNG2, HNF4A, HNF4G, MAPK7, MEF2D, NFATC2, NR0B2, NR1D1, NR1D2, NR1H2, NR2C1, NR2C2, NR2C2AP, NR2E1, NR2F1, NR2F6, NR4A1, NR4A2, NR5A1, NRBP1, POMC, PPARA, PPARD, PPARG, RARA, RARB, RARG, RORA, RORB, RORC, RXRA, RXRG, THRA, THRB, VDR	0.6364	NR4A1
*QC*9	Count: 5		
	APOB, C1QA, C1QB, C1QC, CALR	0.7000	CALR

The human proteins involved in the quasi-cliques are reported along with the densities of the quasi-cliques and the overlapping human proteins with the second quasi-biclique.

### GO and Pathway Analyses of Quasi-Cliques

Subsequently we further analyze the quasi-cliques found (Tables 2 and 3) using Gene Ontology (GO) and pathway based studies. Let us denote the 9 quasi-cliques of Table 2 and 3 by 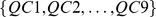 respectively. For the GO and pathway analyses, the web-based tool DAVID (http://david.abcc.ncifcrf.gov/) has been used. Table 4 shows the top few significant GO and KEGG pathway terms for the 9 quasi-cliques along with the significance p-values. It is evident from the table that for all the quasi-cliques have significant GO and KEGG pathways associated with them, with one exception for 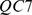 for which no significant KEGG pathway has been found. 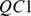 mainly consists of the proteins that function in negative regulation of ubiquitin and participate in proteasome complex whose main function is to degrade unneeded or damaged proteins by proteolysis, a chemical reaction that breaks peptide bonds. The relationship between ubiquitin, proteasome and hepatitis-c have already been reported in literature [28], [29] which involves HCV protein CORE. It may be noticed that the HCV CORE protein belongs to the first quasi-biclique (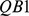 in Table 1, that has overlaps with the quasi-clique 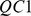. The overlap between 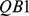 and 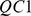 consists of two human proteins PSMB9 and PSME3 and thus they may be considered as possible infection gateway by the HCV proteins CORE (interacts with PSME3), NS3 (interacts with PSMB9) and NS5A (interacts with PSMB9) which belong to quasi-biclique 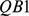, for attacking the proteasome complex.

**Table 4:** The significant important GO terms and KEGG pathways found in the quasi-cliques.

Quasi-clique		Significant GO terms		KEGG Pathway
	Biological Process	Molecular Function	Cellular Component	
*QC*1	negative regulation of ubiquitin-protein ligase activity during mitotic cell cycle	threonine-type endopeptidase activity	proteasome complex	Proteasome
	(p-value: 4.6e-11, 75%)	(p-value: 6.1e-11, 62.5%)	(p-value: 6.4e-14, 87.5%)	(p-value: 3.1e-12, 87.5%)
*QC*2	apoptosis	death domain binding	membrane raft	Apoptosis
	(p-value: 8.9e-14, 85.7%)	(p-value: 5.0e-3, 14.3%)	(p-value: 3.9e-8, 42.9%)	(p-value: 1.1e-10, 57.1%)
	programmed cell death		death-inducing signaling complex	pathways in cancer
	(p-value: 1.1e-13, 85.7%)		(p-value: 5.5e-6, 21.4%)	(p-value: 3.6e-4, 42.9%)
*QC*3	transcription initiation from RNA polymerase II promoter	general RNA polymerase II transcription factor activity	DNA-directed RNA polymerase II, holoenzyme	Basal transcription factors
	(p-value: 6.0e-29, 71.4%)	(p-value: 4.9e-20, 52.4%)	(p-value: 4.1e-30, 76.2%)	(p-value: 1.8e-29, 71.4%)
*QC*4	negative regulation of transcription	enzyme binding	PML body	p53 signaling pathway
	(p-value: 1.0e-8, 87.5%)	(p-value: 1.2e-3, 50.0%, )	(p-value: 1.6e-7, 50.0%)	(p-value: 1.0e-3, 37.5%)
				Chronic myeloid leukemia
				(p-value: 1.3e-3, 37.5%)
*QC*5	protein kinase cascade	protein tyrosine kinase activity	dendrite	Jak-STAT signaling pathway
	(p-value: 2.8e-9, 87.5%)	(p-value: 3.3e-3, 37.5%)	(p-value: 7.4e-2, 25.0%)	(p-value: 4.9e-7, 75.0%)
				Pancreatic cancer
				(p-value: 9.1e-5, 50.0%)
*QC*6	oxidation reduction	procollagen-lysine 5-dioxygenase activity	endoplasmic reticulum	Lysine degradation
	(p-value: 1.0e-4, 100.0%)	(p-value: 1.1e-7, 75.0%)	(p-value: 5.6e-3, 75.0%)	(p-value: 6.0e-7, 100.0%)
*QC*7	anaphase-promoting complex-dependent proteasomal ubiquitin-dependent protein catabolic process	structural constituent of ribosome	cytosolic small ribosomal subunit	
	(p-value: 2.3e-5, 60.0%)	(p-value: 2.6e-2, 40.0%)	(p-value: 1.2e-2, 40.0%)	–
			proteasome complex	
			(p-value: 1.9e-2, 40.0%)	
*QC*8	regulation of transcription, DNA-dependent	steroid hormone receptor activity	nuclear lumen	Pathways in cancer
	(p-value: 4.3e-27, 84.4%)	(p-value: 6.1e-75, 73.3%)	(p-value: 1.4e-3, 20.0%)	(p-value: 1.7e-5, 20.0%)
		transcription factor activity	transcription factor complex	PPAR signaling pathway
		(p-value: 7.7e-37, 84.4%)	(p-value: 4.7e-3, 8.9%)	(p-value: 1.3e-4, 11.1%)
*QC*9	protein maturation	carbohydrate binding	extracellular space	Prion diseases
	(p-value: 2.8e-6, 80.0%)	(p-value: 5.4e-2, 40.0%)	(p-value: 5.9e-4, 80.0%)	(p-value: 1.4e-4, 60.0%)
	humoral immune response mediated by circulating immunoglobulin			Complement and coagulation cascades
	(p-value: 3.0e-5, 60.0%)			(p-value: 5.4e-4, 60.0%)
Quasi-clique		Significant GO terms		KEGG Pathway
	Biological Process	Molecular Function	Cellular Component	
*QC*1	negative regulation of ubiquitin-protein ligase activity during mitotic cell cycle	threonine-type endopeptidase activity	proteasome complex	Proteasome
	(p-value: 4.6e-11, 75%)	(p-value: 6.1e-11, 62.5%)	(p-value: 6.4e-14, 87.5%)	(p-value: 3.1e-12, 87.5%)
*QC*2	apoptosis	death domain binding	membrane raft	Apoptosis

The significant terms are mentioned along with their significance p-values and percentage of proteins associated with each term. DAVID online tool has been used to perform the significance tests.

The quasi-clique 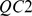 contains 14 human proteins mostly involved in apoptosis and programmed cell death. Also it is interesting that a significant GO-CC term for these proteins is death-inducing signaling complex. Further, these proteins also participate in the KEGG pathway apoptosis as well as pathways in cancer. These evidences suggest strongly that the human proteins involved in this quasi-clique have direct or indirect relationship to cancer diseases. The quasi-biclique 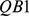 (involving the viral proteins CORE, NS5A and NS3) overlaps with 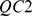 on three human proteins TRADD (interacts with CORE and NS5A), TRAF2 (interacts with CORE and NS5A) and VIM (interacts with CORE and NS3). This suggests that attack by HCV proteins CORE, NS5A and NS3 may lead to cancer through apoptosis and the main gateway host proteins responsible for that are TRADD, TRAF2 and VIM.

The 23 host proteins in quasi-clique 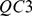 are mainly transcription factors (Table 4). Although the quasi-biclique 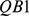 only overlaps with 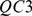 on two host proteins HNRNPK and TBP, it suggests that the viral proteins in 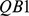 may indirectly interact with many transcription factor proteins and thus may cause their malfunctioning. This may lead to breakdown of the overall setup of normal regulatory roles of these transcription factors causing serious infectious behavior.

Most of the host proteins in the quasi-clique 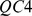 negatively regulate transcription and participate in enzyme binding. It can be noticed that many of these proteins are part of PML bodies, which is a class of nuclear body and they react against SP100 auto-antibodies (PML, promyelocytic leukemia). This is in fact also evident from the pathway analysis which finds two significant KEGG pathways, namely p53 signaling pathway and chronic myeloid leukemia. For the quasi-biclique 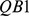 the viral gateway to these host proteins is TP53, a membrane protein that is common for 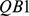 and 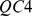. Noticeably, all the viral proteins of 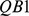, i.e., CORE, NS5A and NS3 interact with TP53 to get entrance. This infection may ultimately lead to chronic myeloid leukemia [30].

The quasi-clique 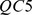 contains host proteins with mainly kinase activities. Two significant KEGG pathways namely JAK-STAT signaling pathway and pancreatic cancer, have been identified in this quasi-clique. This suggests that the HCV proteins in 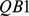 interact with the host proteins in 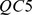 through the common host proteins JAK1, STAT1 and STAT3 leading to pancreatic cancer. Moreover, JAK-STAT system transmits information from chemical signals outside the cell, through the cell membrane. Therefore the proteins involved in 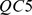 are possibly involved in transferring and propagating the infection to the other cells. A study in [31] has already established the involvement of HCV in JAK-STAT signaling pathway.

The quasi-cliques 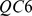 through 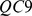 (Table 3) overlap with the quasi-biclique 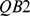, which consists of 5 viral proteins E1, E2, NS2, NS4A, and NS5B and 10 host proteins. 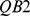 overlaps with 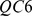 with the host protein SETD2. The most significant GO terms associated with the human proteins in 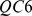 in BP, MF and CC categories are oxidation reduction, procollagen-lysine 5-dioxygenase activity and endoplasmic reticulum, respectively. The most significant KEGG pathway associated with these proteins is Lysine degradation, where all the 4 proteins in 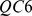 are involved. The association of HCV NS2 protein and lysine degradation is also reported in [32].


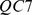
 overlaps 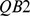 with the host protein UBQLN1. 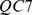 also has proteasomal acitivities 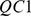, and as discussed before the host proteins in this functional module are involved in hepatitis C infection. However, we could not find any significant pathway for 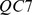.


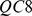
 is the largest quasi-clique that we have found in the present study. This functional module consists of 45 host proteins which are mostly transcription factors. The infection gateway to this module is NR4A1, which is the only common host protein for 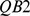 and 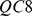. Interestingly, all the five viral proteins in 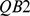 interact with NR4A1, and the CORE protein, which is a part of 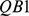 also interacts with NR4A1. This observation suggests that NR4A1 serves as a very important gateway to this transcription factor complex. Any disturbance to this module for viral infection may lead to malfunctioning of normal gene regulatory network, and this in turn can result in various types of cancer (as the pathway study reveals). Our pathway study also reveals another significant pathway, namely PRAR signaling pathway, which is also shown to be associated with HCV infection in recent studies [33].

The quasi-clique 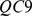 that consists of 5 host proteins which have been found to be associated with protein maturation and humoral immune response mediated by circulating immunoglobulin. Thus these proteins are highly responsible for maintaining the immunity system inside human body. 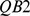 and 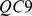 has one common host protein CALR, and hence this protein serves as a gateway of attack to the immunity system by HCV. The viral proteins E1 and E2 (envelop proteins), which are major players in all events required for virus entry into target cells interact with CALR and start attacking the immunity system. This may ultimately lead to many prion diseases (as revealed through pathway analysis).

The GO and pathway analyses of the identified quasi-cliques in human protein interaction network reveals that the host proteins involved in these functional modules have high degree of functional similarities. Moreover, as discussed, HCV attacks that go through these quasi-cliques may lead malfunctioning of regulatory and immunity system in targeted cells and may lead to different types of disease including various types of cancers.

### Mining Quasi-Bicliques in Human Protein-Disease Association Network

To study the disease association with the host proteins in the identified quasi-cliques for finding possible pathway of pathogenesis leading to various diseases, we apply our quasi-biclique finding algorithm on the human gene-disease association network. Note that while finding the quasi-bicliques, we executed the quasi-biclique finding method on 9 different bipartite graphs, corresponding to the 9 quasi-cliques. Each of these graphs contain the human proteins from the corresponding quasi-clique, and all the diseases. The *γ* value is set to 0.7, so that each identified quasi-biclique has density of at least 0.7. Out of the nine quasi-cliques, we found four quasi-cliques 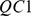, 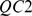, 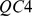 and 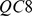 which have overlap with the obtained quasi-bicliques on protein-disease association networks. These quasi-bicliques, termed as 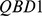, 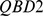, 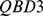, 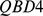 are reported in Table 5. In each quasi-biclique in human protein-disease association network, two human proteins have been found to overlap with the corresponding quasi-cliques. These proteins, thus can be considered as gateways to the diseases. 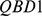 has overlap with 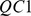 with two proteins PSMB8 and PSMB9 which are associated with five different diseases. 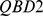 overlaps with 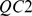 with two host proteins TNFRSF1A and TNFRSF1B and these proteins are highly associated with 12 diseases. The quasi-clique 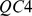 and the quasi-biclique 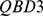 has two common proteins 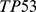 and 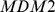 which are connected two 9 diseases including various types of cancer. Two proteins TGFR and MDM2 are common to 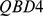 and 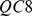 and these proteins have association with 5 diseases which are mainly different cancer types. Interestingly MDM2 belongs to both 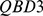 and 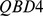.

**Table 5:** Quasi-bicliques found for human protein-disease association network corresponding to four quasi-cliques.

Quasi-biclique	Corresponding *QC*	Human proteins	Diseases	Density
*_QBD_*_1_	*QC*1	Count: 2	Count: 5	
		PSMB8, PSMB9	Graves disease, diabetes (type 1), interferon response, psoriasis, malaria; hypoglycemia; hyperparasitemia	0.7000
*_QBD_*_2_	*QC*2	Count: 2	Count: 12	
		TNFRSF1A, TNFRSF1B	Crohn's disease, ulcerative colitis, cystic fibrosis, Lupus, Rheumatoid Arthritis, diabetes (type 2), amyloidosis, breast cancer, Tumor necrosis factor receptor-associated periodic syndrome, bone density, bone mass, obesity	0.7083
*_QBD_*_3_	*QC*4	Count: 2	Count: 9	
		TP53, MDM2	DNA Damage | Lung Neoplasms, B-Cell Chronic Lymphocytic Leukemia, bladder cancer, breast cancer, colorectal cancer, endometrial cancer, liver cancer, lung cancer, stomach cancer	1.000
*_QBD_*_4_	*QC*8	Count: 2	Count: 5	
		EGFR, MDM2	colorectal cancer, lung cancer, Acute Coronary Syndrome, Breast Neoplasms Carcinoma | Non-Small-Cell Lung | Exanthema | Lung Neoplasms	0.7000

The human proteins and diseases associated with each quasi-biclique are reported along with the densities of the quasi-bicliques.

### Analyses of Gateway Proteins

Previous results and discussions have pointed out two types of gateway proteins, one set acts as the gateway to the host cellular mechanism for the viral proteins, and the second set consists of the host proteins that have high degree of association to different kinds of diseases. The first set *VH* (Viral-Host) contains 15 host proteins: PSME3, TP53, TBP, TRADD, STAT3, HNRNPK, NR4A1, SETD2, PSMB9, TRAF2, STAT1, CALR, JAK1, VIM and UBQLN1 (Tables 2 and 3). The second set *HD* (Human-Disease) contains 7 host proteins PSMB8, PSMB9, TNFRSF1A, TNFRSF1B, TP53, MDM2 and EGFR. The results reveal that HCV infection pathogenesis should propagate through the proteins in *VH* and *HD* sets, and thus these proteins play extremely important role during viral infection. Specially, the proteins in the set *VH* are responsible for the initiation of the infection process. First we compare the average degrees of gateway and non-gateway proteins and found that average degree of gateway proteins is 21.6364, whereas the average degree of non-gateway proteins is 4.2295. The difference is statistically significant as per Wilcoxon's rank sum test (p-value: 1.3006e-09). This suggests that the viral proteins tend to attack high-degree host proteins for initiating infection. Moreover, to test whether these proteins have some unique features, we investigate for their GO (BP) and pathway enrichment (Table 6). It is evident from the table that the significant GO-BP terms mostly involved in apoptosis and programmed cell death which indicates that the targeted host proteins are highly associated with the process of cell death. Moreover significant pathways suggest that HCV infection ultimately lead to various cancer types including pancreatic cancer which is already established in a recent study [53].

**Table 6:** Significant GO-BP and KEGG pathway terms for viral-human gateway proteins.

Significant GO-BP terms
cytokine-mediated signaling pathway (p-value: 3.7e-5)
regulation of apoptosis (p-value: 5.2e-5)
regulation of programmed cell death (p-value: 5.5e-5)
regulation of cell death (p-value: 5.6e-5)
positive regulation of macromolecule metabolic process (p-value: 7.4e-5)
**Significant KEGG pathways**
Pancreatic cancer (p-value: 5.5e-4)
Pathways in cancer (p-value: 5.6e-3)

The significant terms are mentioned along with their significance p-values. DAVID online tool has been used to perform the significance tests.

## Conclusions

In this article a system-wide study has been made for identifying possible infection pathway of hepatitic C virus. For this purpose, quasi-bicliques in HCV-human protein interaction network are mapped onto quasi-cliques in human protein interaction network. Subsequently, the quasi-cliques are mapped onto human protein-disease association networks. Hierarchical clustering based quasi-clique and quasi-biclique mining algorithms have been proposed in this context. The quasi-cliques that overlap with the quasi-bicliques in HCV-human protein interaction network have been found to contain host proteins highly associated in various disease pathways including different cancer types. Many of the diseases have evidence in literature for their connection with HCV infection. Further, the gateway proteins, i.e., the proteins which are mainly targeted by HCV proteins to disturb the host cellular mechanisms, are identified. These gateway proteins have been found to have high degrees in human interactome compared to the other virus-targeted proteins. Moreover, the gateway proteins are tested for GO-BP enrichment and pathway enrichment, and these analyses reveal that these proteins are highly involved in apoptosis and programmed cell death leading to various cancer types.

## Supporting Information

File S1 Excel file containing hepatitis C-human protein-protein interaction network.
(XLS)

File S2Excel file containing human protein interactomes induced by quasi-bicliques 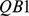 and 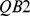 of hepatitis C-human protein interaction network.(XLS)

File S3 Excel file containing human disease-gene association network.
(XLS)

File S4 Excel file containing all quasi-cliques and quasi-bicliques.
(XLS)

## References

[1] Panchenko A, Przytycka T (2008) Protein-protein Interactions and Networks: Identification, Computer Analysis, and Prediction, volume 9. London: Springer-Verlag.

[2] Ito T, Chiba T, Ozawa R, Yoshida M, Hattori M, et al (2001) A comprehensive two-hybrid analysis to explore the yeast protein interactome. Proceedings of the National Academy of Sciences, USA 10: 4569–4574. 10.1073/pnas.061034498 PMC31875 11283351

[3] Uetz P, Giot L, Cagney G, Mansfield TA, Judson RS, et al (2000) A comprehensive analysis of protein-protein interactions in *Saccharomyces cerevisiae* . Nature 403: 623–627. 10688190 10.1038/35001009

[4] Rual JF, Venkatesan K, Hao T, Hirozane-Kishikawa T, Dricot A, et al (2005) Towards a proteomescale map of the human protein-protein interaction network. Nature 437: 1173–1178. 16189514 10.1038/nature04209

[5] Stelzl U, Worm U, Lalowski M, Haenig C, Brembeck FH, et al (2005) A human protein-protein interaction network: a resource for annotating the proteome. Cell 122: 957–968. 16169070 10.1016/j.cell.2005.08.029

[6] Gavin AC, Bsche M, Krause R, Grandi P, Marzioch M, et al (2002) Functional organization of the yeast proteome by systematic analysis of protein complexes. Nature 415: 141–147. 11805826 10.1038/415141a

[7] Ho Y, Gruhler A, Heilbut A, Bader GD, Moore L, et al (2002) Systematic identification of protein complexes in *Saccharomyces cerevisiae* by mass spectrometry. Nature 415: 180–183. 11805837 10.1038/415180a

[8] Huang J, Schreiber SL (1997) A yeast genetic system for selecting small molecule inhibitors of protein-protein interactions in nanodroplets. Proceedings of the National Academy of Sciences, USA 94: 13396–13401. 10.1073/pnas.94.25.13396 PMC28315 9391035

[9] Arkin MR, Wells JA (2004) Small-molecule inhibitors of protein-protein interactions: progressing towards the dream. Nature Reviews Drug Discovery 3: 301–317. 15060526 10.1038/nrd1343

[10] Tastan O, Qi Y, Carbonell J, Klein-Seetharaman J (2009) Prediction of interactions between HIV-1 and human proteins by information integration. In: Proceedings of the Pacific Symposium on Biocomputing. pp. 516–527. PMC3263379 19209727

[11] MacPherson JI, Dickerson JE, Pinney JW, Robertson DL (2010) Patterns of HIV-1 Protein Interaction Identify Perturbed Host-Cellular Subsystems. PLoS Comput Biol 6: e1000863+. 20686668 10.1371/journal.pcbi.1000863 PMC2912648

[12] Mukhopadhyay A, Maulik U, Bandyopadhyay S, Eils R (2010) Mining association rules from hivhuman protein interactions. In: Proc. Intl. Conf. con Systems in Medicine and Biology (ICSMB 2010). pp. 344–348.

[13] Maulik U, Bhattacharyya M, Mukhopadhyay A, Bandyopadhyay S (2011) Identifying the immunodeficiency gateway proteins in humans and their involvement in microrna regulation. Mol BioSyst 7: 1842–1851. 21437347 10.1039/c1mb05026e

[14] Mukhopadhyay A, Maulik U, Bandyopadhyay S (2012) A novel biclustering approach to association rule mining for predicting hiv-1–human protein interactions. PLoS ONE 7: e32289. 22539940 10.1371/journal.pone.0032289 PMC3335119

[15] Mukhopadhyay A, Ray S, Maulik U (2014) Incorporating the type and direction information in predicting novel regulatory interactions between hiv-1 and human proteins using a biclustering approach. BMC Bioinformatics 15: 26. 24460683 10.1186/1471-2105-15-26 PMC3922888

[16] Lorenz IC (2010) The hepatitis C virus nonstructural protein 2 (NS2): An up-and-coming antiviral drug target. Viruses 2: 1635–1646. 21994698 10.3390/v2081635 PMC3185728

[17] Tripathi LP, Kataoka C, Taguwa S, Moriishi K, Mori Y, et al (2010) Network based analysis of hepatitis C virus Core and NS4B protein interactions. Mol BioSyst 6: 2539–2553. 20953506 10.1039/c0mb00103a

[18] Popescu CI, Callens N, Trinel D, Roingeard P, Moradpour D, et al (2011) NS2 protein of hepatitis C virus interacts with structural and non-structural proteins towards virus assembly. PLoS Pathog 7: e1001278. 21347350 10.1371/journal.ppat.1001278 PMC3037360

[19] Zheng S, Tansey WP, Hiebert SW, Zhao Z (2011) Integrative network analysis identifies key genes and pathways in the progression of hepatitis C virus induced hepatocellular carcinoma. BMC Med Genomics 4: 62. 21824427 10.1186/1755-8794-4-62 PMC3212927

[20] Kwofie SK, Schaefer U, Sundararajan VS, Bajic VB, Christoffels A (2011) HCVpro: Hepatitis C virus protein interaction database. Infect Genet Evol 10.1016/j.meegid.2011.09.001 21930248

[21] Bhattacharyya M, Bandyopadhyay S (2009) Solving maximum fuzzy clique problem with neural networks and its applications. Memetic Computing 1: 281–290.

[22] Bhattacharyya M, Bandyopadhyay S (2012) Bounds on Quasi-Completeness, LNCS, Springer. IWOCA.

[23] Jain AK, Dubes RC (1988) Algorithms for Clustering Data. Englewood Cliffs, NJ: Prentice-Hall.

[24] Mukhopadhyay A, Maulik U, Bandyopadhyay S (2007) Multiobjective genetic fuzzy clustering of categorical attributes. In: Proc. 10th International Conference on Information Technology (ICIT 2007). pp. 74–79.

[25] Mukhopadhyay A, Maulik U, Bandyopadhyay S (2010) On biclustering of gene expression data. Current Bioinformatics 5: 204–216.

[26] de Chassey B, Navratil V, Tafforeau L, Hiet MS, Aublin-Gex A, et al (2008) Hepatitis C virus infection protein network. Mol Syst Biol 4: 230. 18985028 10.1038/msb.2008.66 PMC2600670

[27] Zhang Y, De S, Garner JR, Smith K, Wang SA, et al (2010) Systematic analysis, comparison, and integration of disease based human genetic association data and mouse genetic phenotypic information. BMC Medical Genomics 21. 10.1186/1755-8794-3-1 PMC2822734 20092628

[28] Osna NA, White RL, Krutik VM, Wang T, Weinman SA, et al (2008) Proteasome activation by hepatitis C core protein is reversed by ethanol-induced oxidative stress. Gastroenterology 134: 2144–2152. 18549882 10.1053/j.gastro.2008.02.063 PMC2517112

[29] Suzuki R, Moriishi K, Fukuda K, Shirakura M, Ishii K, et al (2009) Proteasomal turnover of hepatitis C virus core protein is regulated by two distinct mechanisms: a ubiquitin-dependent mechanism and a ubiquitin-independent but PA28gamma-dependent mechanism. Journal of Virology 83: 2389–2392. 19091860 10.1128/JVI.01690-08 PMC2643730

[30] Klco JM, Geng B, Brunt EM, Hassan A, Nguyen TD, et al (2010) Bone marrow biopsy in patients with hepatitis C virus infection: spectrum of findings and diagnostic utility. American Journal of Hematology 85: 106–110. 20095034 10.1002/ajh.21600

[31] Zhang L, Jilg N, Shao RX, Lin W, Fusco DN, et al (2011) IL28B inhibits hepatitis C virus replication through the JAK-STAT pathway. Journal of Hepatology 55: 289–298. 21147189 10.1016/j.jhep.2010.11.019 PMC3068235

[32] Welbourn S, Jirasko V, Breton V, Reiss S, Penin F, et al (2009) Investigation of a role for lysine residues in non-structural proteins 2 and 2/3 of the hepatitis C virus for their degradation and virus assembly. Journal of General Virology 90: 1071–1080. 19264595 10.1099/vir.0.009944-0

[33] Negro F (2009) Peroxisome Proliferator-Activated Receptors and Hepatitis C virus-induced insulin resistance. PRAR Research 10.1155/2009/483485 PMC2614087 19132131

[34] Kim BK, Choi YS, Park YH, Lee SU (2011) Interferon-alpha-induced destructive thyroiditis followed by Graves' disease in a patient with chronic hepatitis C: a case report. J Korean Med Sci 26: 1638–1641. 22148004 10.3346/jkms.2011.26.12.1638 PMC3230027

[35] Chen LK, Chou YC, Tsai ST, Hwang SJ, Lee SD (2005) Hepatitis C virus infection-related Type 1 diabetes mellitus. Diabet Med 22: 340–343. 15717885 10.1111/j.1464-5491.2005.01412.x

[36] Giordanino C, Ceretto S, Bo S, Smedile A, Ciancio A, et al (2012) Type 2 diabetes mellitus and chronic hepatitis C: which is worse? Results of a long-term retrospective cohort study. Dig Liver Dis 44: 406–412. 22245505 10.1016/j.dld.2011.12.003

[37] Tsuge M, Fujimoto Y, Hiraga N, Zhang Y, Ohnishi M, et al (2011) Hepatitis C virus infection suppresses the interferon response in the liver of the human hepatocyte chimeric mouse. PLoS ONE 6: e23856. 21886832 10.1371/journal.pone.0023856 PMC3160317

[38] Yamamoto T, Katayama I, Nishioka K (1995) Psoriasis and hepatitis C virus. Acta Derm Venereol 75: 482–483. 8651030 10.2340/0001555575482483

[39] Ouwe-Missi-Oukem-Boyer O, Ndouo FS, Ollomo B, Mezui-Me-Ndong J, Noulin F, et al (2011) Hepatitis C virus infection may lead to slower emergence of P. falciparum in blood. PLoS ONE 6: e16034. 21249226 10.1371/journal.pone.0016034 PMC3018426

[40] Salcedo-Mora X, Mate J, Medina J, Nam Cha SJ, Gisbert JP, et al (2006) Chronic hepatitis C and Crohn's disease: nosocomial infection treatment with PEG-interferon plus ribavirin. Digestion 73: 210–214. 16883071 10.1159/000094787

[41] Hou JK, Velayos F, Terrault N, Mahadevan U (2010) Viral hepatitis and inflammatory bowel disease. Inflamm Bowel Dis 16: 925–932. 20480515 10.1002/ibd.21284

[42] Perlemuter G, Cacoub P, Sbai A, Hausfater P, Thibault V, et al (2003) Hepatitis C virus infection in systemic lupus erythematosus: a case-control study. J Rheumatol 30: 1473–1478. 12858443

[43] Ferri C, Sebastiani M, Antonelli A, Colaci M, Manfredi A, et al (2012) Current treatment of hepatitis C-associated rheumatic diseases. Arthritis Res Ther 14: 215. 22731694 10.1186/ar3865 PMC3446515

[44] de Oliveria Andrade LJ, D'Oliveira A, Melo RC, De Souza EC, Costa Silva CA, et al (2009) Association between hepatitis C and hepatocellular carcinoma. J Glob Infect Dis 1: 33–37. 20300384 10.4103/0974-777X.52979 PMC2840947

[45] Su FH, Chang SN, Chen PC, Sung FC, Su CT, et al (2011) Association between chronic viral hepatitis infection and breast cancer risk: a nationwide population-based case-control study. BMC Cancer 11: 495. 22115285 10.1186/1471-2407-11-495 PMC3261833

[46] Emilia G, Luppi M, Ferrari MG, Barozzi P, Marasca R, et al (1997) Hepatitis C virus-induced leuko-thrombocytopenia and haemolysis. J Med Virol 53: 182–184. 9334931 10.1002/(sici)1096-9071(19971003)53:2<182::aid-jmv12>3.0.co;2-l

[47] Calle EE, Rodriguez C, Walker-Thurmond K, Thun MJ (2003) Overweight, obesity, and mortality from cancer in a prospectively studied cohort of U.S. adults. N Engl J Med 348: 1625–1638. 12711737 10.1056/NEJMoa021423

[48] Mohamed AA, Loutfy SA, Craik JD, Hashem AG, Siam I (2011) Chronic hepatitis c genotype-4 infection: role of insulin resistance in hepatocellular carcinoma. Virol J 8: 496. 22044490 10.1186/1743-422X-8-496 PMC3218090

[49] Omland LH, Farkas DK, Jepsen P, Obel N, Pedersen L (2010) Hepatitis C virus infection and risk of cancer: a population-based cohort study. Clin Epidemiol 2: 179–186. 20865115 10.2147/clep.s10193 PMC2943195

[50] Schiefke I, Fach A, Wiedmann M, Aretin AV, Schenker E, et al (2005) Reduced bone mineral density and altered bone turnover markers in patients with non-cirrhotic chronic hepatitis B or C infection. World J Gastroenterol 11: 1843–1847. 15793878 10.3748/wjg.v11.i12.1843 PMC4305888

[51] Nanda KS, Ryan EJ, Murray BF, Brady JJ, McKenna MJ, et al (2009) Effect of chronic hepatitis C virus infection on bone disease in postmenopausal women. Clin Gastroenterol Hepatol 7: 894–899. 19558999 10.1016/j.cgh.2009.01.011

[52] Butt AA, Xiaoqiang W, Budoff M, Leaf D, Kuller LH, et al (2009) Hepatitis C virus infection and the risk of coronary disease. Clin Infect Dis 49: 225–232. 19508169 10.1086/599371 PMC3077953

[53] El-Serag HB, Engels EA, Landgren O, Chiao E, Henderson L, et al (2009) Risk of hepatobiliary and pancreatic cancers after hepatitis c virus infection: A population-based study of u.s. veterans. Hepatology 49: 116–123. 19085911 10.1002/hep.22606 PMC2719902

